# A Quantitative Trait Locus for Reduced Microglial *APOE* Expression Associates with Reduced Cerebral Amyloid Angiopathy

**DOI:** 10.1101/2025.03.10.25323519

**Published:** 2025-03-13

**Authors:** Michael E. Belloy, Jonathan Graff-Radford, Michael D. Greicius

**Affiliations:** 1 NeuroGenomics and Informatics Center, Washington University School of Medicine, St.Louis, MO, USA; 2 Department of Neurology, Washington University School of Medicine, St.Louis, MO, USA; 3 Departments of Neurology, Mayo Clinic, Rochester, MN, USA; 4 Department of Neurology and Neurological Sciences, Stanford University School of Medicine, Stanford, CA, USA

## Abstract

The *Apolipoprotein E (APOE)* e4 and e2 alleles are respectively the most risk increasing and risk decreasing, common genetic risk factors for Alzheimer’s disease (AD)^1,2^. They strongly affect Aβ burden in the brain parenchyma^1^, a core hallmark of AD, but also at the level of the brain vasculature, i.e. cerebral amyloid angiopathy (CAA)^1,3^, which in turn relates to increased risk for amyloid-related imaging abnormalities (ARIA) in APOE*4 carriers when receiving anti-Aβ antibody treatments^4^. This makes *APOE* a highly pursued AD drug target. A crucial question in the field is whether it would be beneficial to either increase or decrease *APOE* (particularly APOE*4) levels^5^. The answer from rodent work appears to converge on “decreasing *APOE* levels”^5–7^, with initial human studies supporting this^5,8,9^. Human genetic evidence however remains scarce and new insights are crucially needed to support clinical translation. Shade et al. 2024 conducted the largest to date genome-wide association study (GWAS) of various neuropathological traits, identifying a variant protective of CAA in the *APOE* locus independent of APOE*4 and APOE*2 genotypes^10^. Downstream analyses suggested this signal links to the nearby *APOC2* gene through local effects on methylation. We applaud the authors on their timely, relevant, and well-conducted study. Here, we extend on these findings, highlighting there is compelling evidence that their genetic signal for reduced CAA relates to an effect on reduced microglial *APOE* expression, which would importantly support the evidence in favor of “decreasing *APOE* levels” and further herald this promising therapeutic avenue, not just for AD, but also for CAA. We additionally provide complimentary results regarding this locus’ association with CAA and AD risk from analyses that we conducted parallel to Shade et al. 2024.

## Introduction

Shade et al performed GWAS of neuropathological traits across the National Alzheimer’s Coordinating Center (NACC), the Religious Orders Study and Rush Memory and Aging Project (ROSMAP), and the Adult Changes in Thought (ACT) study. Their CAA GWAS identified rs7247551 at the *APOE* locus after conditioning on APOE*4 and APOE*2 genotypes (Table.1). They further conducted genetic colocalization analyses with expression quantitative trait locus (e)(QTL) data in 48 tissues (including brain) from GTEx and in brain dorsolateral prefrontal cortex (DLPFC) from ROSMAP, as well as methylation (m)QTL data in DLPFC from ROSMAP. While their CAA signal colocalized with *APOC2* eQTLs in multiple brain tissues in GTEx, it did not in ROSMAP. In fact, their top variant, rs7247551, had opposite effects on *APOC2* expression in brain tissues across GTEx and ROSMAP (and was only nominally associated in the latter, P=0.00013). There was not an *APOE* eQTL effect in brain for rs7247551 in either dataset. They did observe colocalization with DLPFC mQTLs near *APOC2*, with 2 implicated CpG sites also associating with CAA and APOC2 expression, yet also noting that *APOC2* expression was not significantly associated with the severity of CAA in ROSMAP (P=0.089). Across these observations and incongruent eQTL results, they reasonably concluded that *APOC2* and related methylation may be the culprit in their CAA genetic signal.

However, earlier this year, Fujita et al. presented a large single-nucleus RNAseq eQTL resource (n=424) in ROSMAP DLPFC (largely overlapping the bulk DLPFC eQTL data evaluated by Shade et al.)^11^. Strikingly, they found a microglia-specific eQTL overlapping the locus identified by Shade et al. Their top variant, rs2288911, is in strong linkage disequilibrium (LD) with rs7247551 (R2=0.98). rs2288911 lies upstream, 5Kb closer to *APOE*, within a site with marks for microglia-specific active enhancers (H3K27ac) and promoters (H3K4me3), as well as evidence for chromatin interactions with *APOE* (PLAC-seq) (cf. Fig.4d in Fujita et al.). They also evaluated associations of rs2288911 with different traits in ROSMAP, highlighting an association with CAA independent of APOE*4 (P=9.9e-6). The rs2288911*T allele, correlated with the protective rs7247551*G allele in Shade et al., was associated with decreased *APOE* expression in microglia and decreased CAA risk.

## Results & Discussion

**Figure 1.**
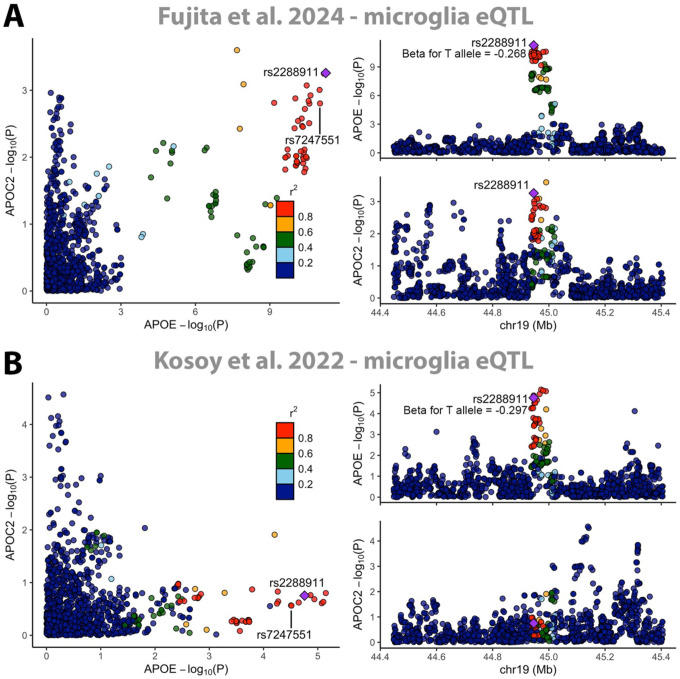
Rs2288911 and rs7247551 are microglia expression quantitative trait loci for *APOE*, not *APOC2*. Locus comparison (left) and locus zoom (right) plots indicate that the two SNPs are microglia eQTLs for *APOE* and *APOC2* in two independent datasets: **(A)** Fujita et al. 2024 (n=424) and **(B)** Kosoy et al. 2022 (n=400). Color coding of variants/dots indicates LD (R2) with rs2288911, marked by purple diamonds (R v4.2.1, locuscomparer library). Plots only display variants present in both datasets (93% overlap within depicted 1Mb range). rs2288911 was identified in Fujita et al. as the lead microglia-specific eQTL for *APOE* and is in strong LD with rs7247551 identified by Shade et al. (R2=0.98), who related its effect to *APOC2* and local methylation. rs2288911 shows only a nominal association with microglial *APOC2* expression in (A) and none in (B), indicating it is not an eQTL for *APOC2*. Conversely, it is strongly associated with *APOE* expression in both datasets with consistent beta coefficients that suggest close to 30% reduced microglia *APOE* expression for a single T allele. *APOE* eQTL signals across (A) and (B) colocalized with PP4=0.94 (R v4.2.1, coloc library). Abbreviations: expression quantitative trait locus, eQTL; Linkage disequilibrium, LD; Colocalization poster probability for hypothesis 4: association with both traits and a shared variant, PP4.

A new inspection of the microglia eQTL data from Fujita et al. illustrates that rs2288911 is an eQTL for *APOE* but not *APOC2* (Fig.1A). This observation replicates in a prior, independent microglia eQTL meta-analysis (n=400) by Kosoy et al. (Fig.1B)^12^, further corroborating that *APOE* expression in microglia, rather than *APOC2*, is the likely mechanism behind this CAA genetic signal. However, it should be noted that rs2288911 appears to be an equally significant eQTL for *APOC1* (Fig.S1), which is the closest downstream gene from *APOE*. Co-regulation of nearby genes is common, and there is to our knowledge no literature implicating *APOC1* in amyloid or CAA pathology, such that *APOE* remains the most likely candidate gene, although future work should consider a potential role of *APOC1*.

Unbeknownst to Shade et al., the earlier Fujita et al. study inspired us to look at the association of rs2288911 with CAA and AD risk in large genetic datasets from the Alzheimer’s Disease Genetics Consortium (ADGC), Alzheimer’s Disease Sequencing Project (ADSP), and UK Biobank (UKB). We leveraged our established pipelines and state-of-the-art *APOE* genotype quality control to ensure the most accurate assessment of genetic associations conditioned on APOE*4 and APOE*2^13,14^. We reproduced the Fujita et al. rs2288911*T-CAA association in ROSMAP and additionally replicated this in NACC data overlapping ADGC and ADSP, effectively reproducing the CAA finding in Shade et al. (Table.1). We further added an additional replication attempt in the population-based Mayo Clinic Study of Aging (MCSA, n=801)^15^, where the effect direction was consistent but diminished and non-significant (Table.1). The MCSA primarily follows cognitively unimpaired individuals or those with mild cognitive impairment. Notably, Shade et al. also performed replication analyses in the MAYO brain bank, specifically MC-CAA (n=815)^3^, and obtained a significant replication (P=0.0012) using an alternative CAA phenotype. While some overlap in sample is present, MC-CAA includes only participants that met neuropathological criteria for AD (NINCDS-ADRDA), with Braak stage>3, while MCSA contained fewer advanced cases. However, sensitivity analyses across NACC and ROSMAP did not indicate a stronger effect size at higher Braak stages, marking some unexplained variability in effect estimates (Braak≤3, OR=0.72, 95%CI=[0.60,0.85], P=2.0e-4, n=1,165; Braak>3, OR=0.85, 95%CI=[0.79,0.94], P=7.2e-4, n=3,735). Finally, we assessed the association of rs2288911*T with AD risk (Table.1). It’s crucial to appreciate here that even the slightest LD of variants at the *APOE* locus with APOE*4 or APOE*2 may inflate association results, which is additionally relevant as AD-specific *APOE*-haplotypes may exist. We thus verified that APOE*4 (rs429358) and APOE*2 (rs7412) have very low (though still detectable) LD with rs2288911 in unrelated case-control samples from ADGC and ADSP (respectively R2=0.00223 and R2=0.00249). We then observed that rs2288911*T is associated with reduced AD risk even after conditioning on APOE*4 and APOE*2 (Table.1). This is the first analysis at this scale to provide association results for rs2288911 with AD risk conditioned on APOE*4 and APOE*2. The effect of rs2288911 on AD risk is consistent with the shared genetic contributors to AD and CAA. The weaker signal in AD compared to CAA supports the consensus that CAA and AD are only partially genetically correlated and that there is still important biological distance between the two diseases.

## Conclusion

In summary, Shade et al., Fujita et al., and our group, provide compelling evidence for the association of rs2288911*T or rs7247551*G at the *APOE* locus with lower CAA risk, independent of APOE*2 and APOE*4. Seeking to reconcile findings across these studies, we suggest the most promising mechanism is that the causal variant reduces *APOE* expression in microglia specifically. Our additional analyses confirm the variant is also associated with AD risk in common AD GWAS cohorts, independent of APOE*2 and APOE*4.

Lastly, we suggest that further exploration of this CAA association, as well as additional replication efforts, will be relevant to confirm its robustness. In conclusion, this genetic signal for CAA at the *APOE* locus and the proposed mechanism represent important targets for further experimental validation, which is bound to generate support for the clinical relevance of lowering APOE to protect against CAA and AD.

## Methods

All genetic association analyses were performed in subjects of European ancestry. CAA risk analyses were performed including unrelated subjects using ordinal regression on the same ordinal CAA outcome measure as in Shade et al. (none<mild<moderate<severe), adjusting for age, sex, clinical diagnosis (including cognitively normal, mild cognitive impairment, Alzheimer’s disease, or other dementia), APOE*4 dosage and APOE*2 dosage as categorical covariates, technical covariates, and the first 5 genetic principal components. AD risk analyses in the Alzheimer’s Disease Genetics Consortium (ADGC) and Alzheimer’s Disease Sequencing Project (ADSP) data were performed including related subjects using linear mixed model regression analyses (LMM-BOLT), adjusting for sex, APOE*4 dosage and APOE*2 dosage as categorical covariates, technical covariates, and the first 5 genetic principal components (PCs). AD risk analyses in UK Biobank (UKB) were performed including related subjects using linear mixed model regression analyses (LMM-BOLT), adjusting for age (both subject and parental), sex, APOE*4 dosage and APOE*2 dosage as categorical covariates, technical covariates, and the first 20 genetic PCs.

Expression quantitative trait locus (eQTL) colocalization analyses for genes at the *APOE* locus were assessed from (1) Fujita et al. 2024 for single nucleus (microglia + 6 additional cell types) data from dorsolateral prefrontal cortex in ROSMAP (N=424) and (2) Kosoy et al. 2022 for single cell (microglia) data meta-analyzed across multiple brain regions and studies (N=400). QTL genetic colocalization analyses were conducted using the coloc library, R v4.2.1.

## Supplementary Material

Supplement 1

## Figures and Tables

**Table 1.** Association of rs2288911*T with cerebral amyloid angiopathy and Alzheimer’s disease.

Outcome	Cohorts	No. subjects	AD/DEM %	EAF	OR [95%CI]	P-value
CAA risk (ordinal)	Shade et al. 2024 - ROSMAP+NACC+ACT ‡	7,381	70.6%	-	0.81 [0.76, 0.86]	8.00E-12
CAA risk (ordinal)	ROSMAP (overlapping Shade et al. 2024)	847	46.5%	50.1%	0.67 [0.54, 0.83]	2.57E-04
CAA risk (ordinal)	NACC (overlapping Shade et al. 2024)	4,126	84.1%	49.0%	0.85 [0.78, 0.92]	1.07E-04
CAA risk (ordinal)	MCSA (independent of Shade et al. 2024)	801	33.5%	47.3%	0.87 [0.73, 1.05]	0.151
AD risk (logistic)	ADGC+ADSP (case-control)	30,518	51.1%	49.7%	0.97 [0.95, 0.99]	3.24E-03
AD risk (logistic)	UKB (family-proxy & health registry)	438,080	14.0%	50.0%	0.98 [0.95, 1.00]	0.055

All analyses were performed in subjects of European ancestry. CAA risk analyses were performed including unrelated subjects using ordinal regression (R v4.2.1, plor function in MASS library) on the same ordinal CAA outcome measure as in Shade et al. (none<mild<moderate<severe), adjusting for age, sex, clinical diagnosis (including cognitively normal, mild cognitive impairment, Alzheimer’s disease, or other dementia), APOE*4 dosage and APOE*2 dosage as categorical covariates, technical covariates, and the first 5 genetic principal components. AD risk analyses in ADGC+ADSP were performed including related subjects using linear mixed model regression analyses (LMM-BOLT v2.3.6), adjusting for sex, APOE*4 dosage and APOE*2 dosage as categorical covariates, technical covariates, and the first 5 genetic principal components. AD risk analyses in UKB were performed including related subjects using linear mixed model regression analyses (LMM-BOLT v2.3.6), adjusting for age (both subject and parental), sex, APOE*4 dosage and APOE*2 dosage as categorical covariates, technical covariates, and the first 20 genetic principal components. Per standard procedure, effect estimates from LMM-BOLT were recomputed to traditional odds ratios and in case of UKB further adjusted to account for the use of proxy phenotypes. For additional methodological details and genetic quality control procedures, cf. Belloy et al. 2022, 2024^13,14^.

‡ Results for Shade et al. 2024 correspond to rs7247551*G, which is in strong LD with rs2288911*T (R2=0.98).

Abbreviations: cerebral amyloid angiopathy, CAA; Alzheimer’s disease, AD; dementia, DEM, odds ratio, OR; effect allele frequency, EAF; Number, No.; Alzheimer’s Disease Genetics Consortium, ADGC; Alzheimer’s Disease Sequencing Project, ADSP; UK Biobank, UKB; National Alzheimer’s Coordinating Center, NACC; Religious Orders Study and Rush Memory and Aging Project, ROSMAP; Adult Changes in Thought, ACT; Mayo Clinic Study of Aging, MCSA.

## Data Availability

Data used in the CAA and AD risk analyses are available upon application to: dbGaP (https://www.ncbi.nlm.nih.gov/gap/) NIAGADS (https://www.niagads.org/) LONI (https://ida.loni.usc.edu/) AMP-AD knowledge portal / Synapse (https://www.synapse.org/) Rush (https://www.radc.rush.edu/) NACC (https://naccdata.org/) UKB (https://www.ukbiobank.ac.uk/) MCSA (https://www.mayo.edu/research/centers-programs/alzheimers-disease-research-center/data-requests) The specific data repositories and identifiers for ADGC and ADSP data are indicated in Table.S1 of the supplement. Microglia eQTL datasets are available from: Fujita et al. 2024 - AMP-AD knowledge portal / Synapse (https://www.synapse.org/; identifier: syn52335732) Kosoy et al. 2022 - AMP-AD knowledge portal / Synapse (https://www.synapse.org/; identifier: syn26207321)

## References

[1] Belloy M. E., Napolioni V. & Greicius M. D. A Quarter Century of APOE and Alzheimer’s Disease: Progress to Date and the Path Forward. Neuron 101, 820–838 (2019). 30844401 10.1016/j.neuron.2019.01.056 PMC6407643

[2] Belloy M. E. APOE Genotype and Alzheimer Disease Risk Across Age, Sex, and Population Ancestry. JAMA Neurol (2023) doi:10.1001/JAMANEUROL.2023.3599. PMC10628838 37930705

[3] Reddy J. S. Genome-wide analysis identifies a novel LINC-PINT splice variant associated with vascular amyloid pathology in Alzheimer’s disease. Acta Neuropathol Commun 9, (2021). 10.1186/s40478-021-01199-2 PMC8147512 34020725

[4] Filippi M. Amyloid-Related Imaging Abnormalities and β-Amyloid-Targeting Antibodies: A Systematic Review. JAMA Neurology vol. 79 291–304 Preprint at 10.1001/jamaneurol.2021.5205 (2022). 35099507

[5] Vance J. M. Report of the APOE4 National Institute on Aging/Alzheimer Disease Sequencing Project Consortium Working Group: Reducing APOE4 in Carriers is a Therapeutic Goal for Alzheimer’s Disease. Annals of Neurology vol. 95 625–634 Preprint at 10.1002/ana.26864 (2024). 38180638

[6] Xiong M. APOE immunotherapy reduces cerebral amyloid angiopathy and amyloid plaques while improving cerebrovascular function. Sci Transl Med 13, (2021). 10.1126/scitranslmed.abd7522 PMC8128342 33597265

[7] Gratuze M. APOE Antibody Inhibits Aβ-Associated Tau Seeding and Spreading in a Mouse Model. Ann Neurol 91, 847–852 (2022). 35285073 10.1002/ana.26351 PMC9285984

[8] Chemparathy A. APOE loss-of-function variants: Compatible with longevity and associated with resistance to Alzheimer’s disease pathology. Neuron 112, 1110–1116.e5 (2024). 38301647 10.1016/j.neuron.2024.01.008 PMC10994769

[9] Mak A. C. Y. Effects of the absence of apolipoprotein E on lipoproteins, neurocognitive function, and retinal function. JAMA Neurol 71, 1228–1236 (2014). 25111166 10.1001/jamaneurol.2014.2011 PMC4714860

[10] Shade L. M. P. GWAS of multiple neuropathology endophenotypes identifies new risk loci and provides insights into the genetic risk of dementia. Nat Genet (2024) doi:10.1038/s41588-024-01939-9. PMC11549054 39379761

[11] Fujita M. Cell subtype-specific effects of genetic variation in the Alzheimer’s disease brain. Nat Genet 56, 605–614 (2024). 38514782 10.1038/s41588-024-01685-y PMC12288883

[12] Kosoy R. Genetics of the human microglia regulome refines Alzheimer’s disease risk loci. Nature Genetics 2022 54:8 54, 1145–1154 (2022). 35931864 10.1038/s41588-022-01149-1 PMC9388367

[13] Belloy M. E. Challenges at the APOE locus: A robust quality control approach for accurate APOE genotyping. Alzheimers Res Ther 14, (2022). 10.1186/s13195-022-00962-4 PMC8815198 35120553

[14] Belloy M. E. Role of the X Chromosome in Alzheimer Disease Genetics. JAMA Neurol (2024) doi:10.1001/jamaneurol.2024.2843. PMC11385320 39250132

[15] Roberts R. O. The Mayo Clinic Study of Aging: Design and sampling, participation, baseline measures and sample characteristics. Neuroepidemiology 30, 58–69 (2008). 18259084 10.1159/000115751 PMC2821441

